# The Anti-fibrosis drug Pirfenidone modifies the immunosuppressive tumor microenvironment and prevents the progression of renal cell carcinoma by inhibiting tumor autocrine TGF-β

**DOI:** 10.1080/15384047.2022.2035629

**Published:** 2022-02-07

**Authors:** Gang Wang, Xiaowan Zhou, Zengli Guo, Nan Huang, Juan Li, Yanfang Lv, Lulu Han, Wei Zheng, Dandan Xu, Dafei Chai, Huizhong Li, Liantao Li, Junnian Zheng

**Affiliations:** a Cancer Institute, Xuzhou Medical University, Xuzhou, Jiangsu, China; b Center of Clinical Oncology, the Affiliated Hospital of Xuzhou Medical University, Xuzhou, China; c Jiangsu Center for the Collaboration and Innovation of Cancer Biotherapy, Cancer Institute, Xuzhou Medical University, Xuzhou, Jiangsu, China

**Keywords:** Pirfenidone, TGF-β, renal cell carcinoma, epithelial-to-mesenchymal transition, tumor microenvironment (TME), myeloid-derived suppressor cells (MDSC)

## Abstract

Transforming growth factor-β (TGF-β) plays a critical role in regulating cell growth and differentiation. Epithelial to mesenchymal transition (EMT) induced by TGF-β promotes cancer cell migration, invasion, and proliferation. Pirfenidone (5-methyl-1-phenyl-2(1 H)-pyridone, PFD), an approved drug for treating pulmonary and renal fibrosis, is a potent TGF-β inhibitor and found reduced incidence of lung cancer and alleviated renal function decline. However, whether PFD plays a role in controlling renal cancer progression is largely unknown. In the present study, we demonstrated that high TGF-β1 expression was negatively associated with ten-year overall survival of patients with renal cancer. Functionally, blockade of TGF-β signaling with PFD significantly suppressed the progression of renal cancer in a murine model. Mechanistically, we revealed that PFD significantly decreased the expression and secretion of TGF-β both in vitro and in vivo tumor mouse model, which further prevented TGF-β-induced EMT and thus cell proliferation, migration, and invasion. Importantly, the downregulation of TGF-β upon PFD treatment shaped the immunosuppressive tumor microenvironment by limiting the recruitment of tumor-infiltrating MDSCs. Therefore, our study demonstrated that PFD prevents renal cancer progression by inhibiting TGF-β production of cancer cells and downstream signaling pathway, which might be presented as a therapeutic adjuvant for renal cancer.

## 1. Introduction

Renal cell carcinoma (RCC) is the third common malignant tumor in urinary cancers, ranked after prostate cancer and bladder cancer. There were 431,288 people diagnosed and 179,368 patients died with RCC all over the world in 2020.^1^ During the past two decades, the global incidence of RCC has increased by about 2%, and more than 30% of patients with RCC have already metastasized at the time of initial diagnosis.^2^ Radical resection is currently the major strategy for RCC treatment. However, due to the heterogeneity of RCC, local recurrence and distant metastasis are prone to occur after surgery.^3,4^ Moreover, RCC is not sensitive to traditional radiotherapy and chemotherapy.^5^ Although immunotherapy brings new hope for RCC patients, only 5–27% of them get objective responses.^6^ Therefore, it is urgent to explore new strategies for RCC treatment.

Metastasis and invasion are the main causes of death in malignant tumor patients and RCC is no exception. Epithelial-mesenchymal transition (EMT) is a multi-faced process regulated by various cytokines and transcription factors, which play an important role in tumor development and metastasis.^7,8^ The occurrence of EMT leads to the loss of epithelial characters of tumor cells and the transformation into mesenchymal-like cells and thus enhances tumor cell proliferation and motility and decreases cell apoptosis.^9^ In kidney cancer, liver cancer, bladder cancer, breast cancer, cervical cancer, advanced lung cancer, esophageal cancer, nasopharyngeal carcinoma, melanoma, etc., EMT was reported to promote tumor progression by multiple mechanisms.^10–12^ Therefore, targeting the EMT process or its mediators could be a potential strategy for cancer treatment, including RCC.

Transforming growth factor β (TGF-β) is a major inducer and regulator for EMT. Higher TGF-β expression is found in renal cancer tissues as compared to that of normal tissues, and plasma TGF-β is positively related to tumor size and tumor stage in RCC.^13^ Studies have shown that inhibiting TGF-β in renal cancer is beneficial for restricting the progression of RCC. Interfering TGF-β-Smad signaling prevents the invasiveness of RCC and increases the overall survival of RCC patients.^14^ According to literature studies, TGF-β exerts its biological function mainly through TGF-β receptors and their downstream Smad signaling to promote EMT.^15–17^ Hence, finding an inhibitor of TGF-β signaling and EMT could be a promising way to treat RCC.

Pirfenidone (PFD) is a broad-based anti-fibrotic drug,^18,19^ which was approved by the US FDA in October 2014 for the treatment of idiopathic pulmonary fibrosis and renal fibrosis.^20^ Mechanistically, PFD prevents fibrosis progression primarily by inhibiting TGF-β and other inflammatory factors.^21^ In neurofibromatosis, uterine fibroids, malignant glioma, pancreatic cancer, breast cancer, and lung cancer, PFD is proved to mitigate tumor progression by suppressing TGF-β expression and thus TGF-β-induced cancer-associated fibroblast activation and collagen production.^22–28^ Moreover, PFD is found playing an antitumoral role in lung cancer through boosting antitumor immunity.^29,30^ However, whether PFD also plays a role in controlling renal cell carcinoma is unclear. In this study, we demonstrate that PFD treatment significantly prevents autocrine TGF-β production and TGF-β-induced EMT, thus inhibiting cancer cell proliferation, migration, and invasion in renal cell carcinoma through reducing activation of R-Smads and degradation of the endogenous TGF-β signaling antagonist c-Ski. Our study for the first time illustrates that anti-fibrosis drug PFD might be used as an effective therapeutic strategy for treatment of RCC.

## 2. Materials and Methods

### 2.1 Cell lines and Reagents

The human renal cell carcinoma cell lines, 786-O and ACHN, were purchased from the Shanghai Institute of Biochemistry and Cell Biology, China Academy of Sciences (Shanghai, China) and cultured in DMEM and RPMI-1640 medium, containing 10% fetal bovine serum (FBS), 1% penicillin–streptomycin, at a 37°C incubator with 5% CO_2_. The murine renal adenocarcinoma cell line Renca was purchased from Cobioer Biosciences and cultured in RPMI-1640 supplemented with 10% FBS, 100 units/ml penicillin, 100 μg/ml streptomycin, 2 mM L-glutamine, 1 mM NEAA, and 1 mM sodium pyruvate solution. Most of the experiments were carried out when cells were grown to 70% confluence. Dulbecco’s Modified Eagle Medium and RPMI-1640 Medium were purchased from Gibco. DMSO was purchased from SIGMA. PFD was from Selleckchem (#S2907). Recombinant human TGF-β1 (#100-21, Peprotech) was reconstituted in 4 mM HCl containing 1 mg/ml bovine serum albumin (BSA). Anti-E-cadherin mAb (#AB40860, Absci), anti-N-cadherin mAb (#AB21474, Absci, China), anti-c-Ski mAb (ab131134, Abcam), anti-Smad2/3 mAb (#5678, CST), anti-phosphorylated Smad2 mAb (#8828, CST) and anti-GAPDH mAb (#sc-32233, Santa Cruz) were used for Western blot. PE/Cy7 anti-mouse CD45 Antibody (#147704, Biolegend), APC anti-mouse/human CD11b Antibody (#101212, Biolegend) and FITC anti-mouse Gr1 Antibody (#108405, Biolegend) were used for flow cytometry.

### 2.2 Cell proliferation assay

Cell proliferation was detected by the Cell Counting Kit-8 (CCK8) assay kit (DOjinDO, USA). Briefly, 786-O or ACHN cells were seeded into 96-well plates and treated with PFD 12 h later in the absence or presence of 5 ng/ml TGF-β1 for different times (0, 24, 48, 72 h). Subsequently, 10 μl of CCK8 solution was added to each well, and the plates were incubated at 37°C for another 1–2 h. The absorbance at 460 nm was measured with a BioTek Cytation 3 imaging reader.

### 2.3 Cell migration and invasion

Cell migration and invasion was determined by using a Corning Transwell plate with a pore size of 8 μm. For the cell migration assay, 786-O and ACHN cells were resuspended in serum-free medium and seeded in the upper chamber, RPMI containing 20% FBS was used as a chemoattractant in the lower chamber. Every chamber was treated with or without PFD in the absence or presence of TGF-β1. After incubation at 37°C for 24 h, residual cells in the upper chamber were removed with cotton swabs and the transmigrated cells under the chamber membrane were fixed in methanol and stained with crystal violet. Thereafter, images were taken for each chamber with a microscope. For statistics, cells in at least five fields were counted and quantified.

For the cell invasion assay, the upper chambers were first coated with 30 μl Matrigel/well (BD Biosciences). The rest of the procedure was the same as cell migration.

For the wound-healing assay, confluent cells were scratched with a 200 μl micropipette tip. Followed by washing with PBS to remove the scratched cells, 1 ml of serum-free medium with or without 0.5 mg/ml PFD and 5 ng/ml TGF-β1 was added to each well. Scratch areas were imaged using an inverted microscope at 0, 12, 24, and 48 h, respectively (100×, Nikon Corporation, Tokyo, Japan).

### 2.4 RNA preparation and real-time PCR

Total RNA was extracted using a TRIzol reagent (Invitrogen, 15596–026). First strain cDNA was synthesized using the Reverse Transcription System (Vazyme, R211-01). Real-time PCR detection for TGF-β1, TGF-β2, TGF-β3, and GAPDH was performed using an ABI PRISM 7500 Sequence Detection System (New York, USA) with the following program: 30 s at 95°C, followed by 40 cycles at 95°C for 5 s and annealing at 60°C for 34 s. Relative mRNA expression was calculated and normalized by GAPDH.

### 2.5 Enzyme-linked immune-absorbent assay (ELISA)

Cells in whole culture medium were seeded in 96-well plates and incubated for 12 h. Then, the whole culture medium was discarded and changed with serum-free medium containing 0 or 0.5 mg/ml of PFD. The supernatant was collected after centrifugation at 2,000 rpm for 5 min. Secretion of recombinant human TGF-β1 in the supernatant was determined using a commercially available ELISA kit for human TGF-β1 (DKW12-1710-096, Dakewe, China) following the manufacturer’s instruction.

For the mouse TGF-β1 assay, peripheral blood and serum of renal tumor-bearing mice were collected and then detected with the ELISA kit for mouse TGF-β1 (DKW12-2710-096, Dakewe, China) according to the manufacturer’s instruction.

### 2.6 Western blot

Cells were seeded in six-well plates and treated with PFD in the absence or presence of TGF-β. Whole cell lysates were collected with RIPA buffer containing inhibitor cocktails for proteinase and phosphatase. Protein concentration was measured using the BSA protein quantification kit. Equal amount of 30 μg of total protein was loaded into the wells of a 10% SDS-PAGE gel. The proteins were then transferred onto 0.45 μm nitrocellulose membrane and incubated overnight at 4°C with appropriate primary antibodies. After incubation with peroxidase-coupled anti-mouse or anti-rabbit IgG at 37°C for 2 h, membranes were then washed and scanned by the Bio-Rad Imaging System. Representative images had been chosen for presentation. For the original raw gel images of Western blot shown in figures, see Supplementary data.

### 2.7 Animal model

Female BALB/c mice aged 6–8 weeks were purchased from Beijing Vital River Laboratory Animal Technology and housed in pathogen-free conditions with 12-h light and 12-hdark cycles. All animal procedures and protocols (IACUC No. 2015040201) were approved by the Laboratory Animal Ethical Committee of Xuzhou Medical University.

To establish a subcutaneous renal tumor model, mice were inoculated with 5 × 10^5^ Renca cells in 100 μl of saline at the right flank. A week later, the mice-bearing tumors were randomly divided into two groups (5–8 mice each) and treated with saline or 200 mg/kg PFD intraperitoneally once a day. Tumor growth and mouse body weight were monitored and recorded every 3 d until the end of the experiment. The experiment was ended 29 d after inoculation, and tumor tissues were collected for imaging and immunohistochemical staining.

### 2.8 Immunohistochemical staining

After resection, the tumor tissues were fixed in 4% paraformaldehyde overnight. Then, the fixed tumor tissues were embedded with paraffin and sectioned at 5 μm thickness and then stained with anti-Ki67 (ServiceBio #GB111499). Pictures were taken with the Olympus VS120 imaging system, and representative pictures were presented in the manuscript. The percentage of proliferative tumor cells was quantified by the ratio of Ki67-positive tumor cells to total tumor cells in the tumor tissues.

### 2.9 Flow cytometry

At the end of the experiment, the tumor tissues were collected and homogenized to prepare the single-cell suspensions. After filtering with a 70 μm cell strainer and lysing the red blood corpuscles, the cells were stained with 1 μg/ml of PE/Cy7 anti-CD45, APC anti-CD11b, and FITC anti-Gr1 antibodies at 4°C for 20 min, followed by washing with ice-cold PBS for twice. The data was acquired by BD Canto II flow cytometry and analyzed with FlowJo software.

### 2.10 Statistical analysis

Data were processed by Prism GraphPad and presented as means ± SD. All experiments were repeated at least three times, and representative pictures were chosen for presentation. Statistical significance was determined by Student’s *t*-test and two-way multiple-range ANOVA. A *P*-value less than 0.05 was considered statistically significant.

## 3. RESULTS

### 3.1 Expression of TGF-β is negatively correlated to patients’ ten-year survival in renal cell carcinoma

**Figure 1.**
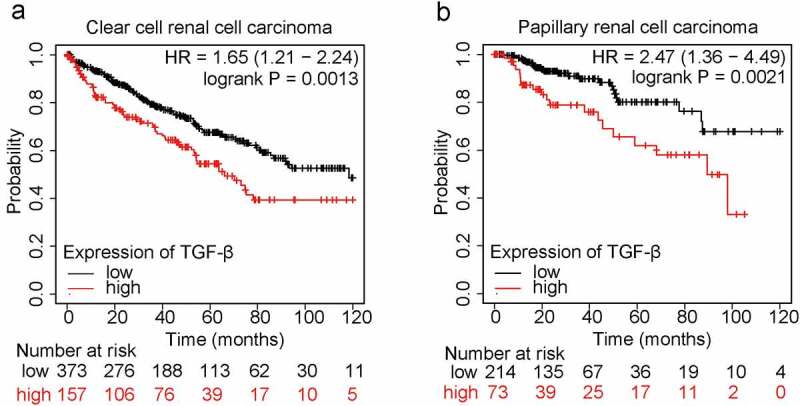
Expression of TGF-β1 is negatively correlated to patients’ survival in renal cell carcinoma.

The TGF-β family members include TGF-β1, TGF-β2, and TGF-β3. Among them, TGF-β1 is the mostexpressed isoform in both developing embryos and adults, which plays a multifunctional role in regulating physiological and pathogenic processes during development (references). Importantly, TGF-β1, as the most common isoform in human cancers (reference PMID: 32710082), is upregulated in renal cancer, head and neck cancer, stomach cancer, etc. To study the functional importance of TGF-β1 in patients with renal cell carcinoma, we analyzed the online database (https://kmplot.com/analysis/) and found that the mRNA expression of TGF-β1 is negatively correlated to the ten-year overall survival of patients in both clear cell renal cell carcinoma and papillary renal cell carcinoma (Figure 1(a,b)). Therefore, TGF-β1 could be an accomplice for the development of renal cancer and a potential target for treatment of renal cancer.

Relation of TGF-β1 mRNA expression to ten-year overall survival of patients with clear cell renal cell carcinoma (A) and papillary renal cell carcinoma (B) were analyzed via excavating the online database (http://kmplot.com).

### 3.2 Blocking of TGF-β signaling by PFD suppresses tumor growth in a murine renal cancer model

**Figure 2.**
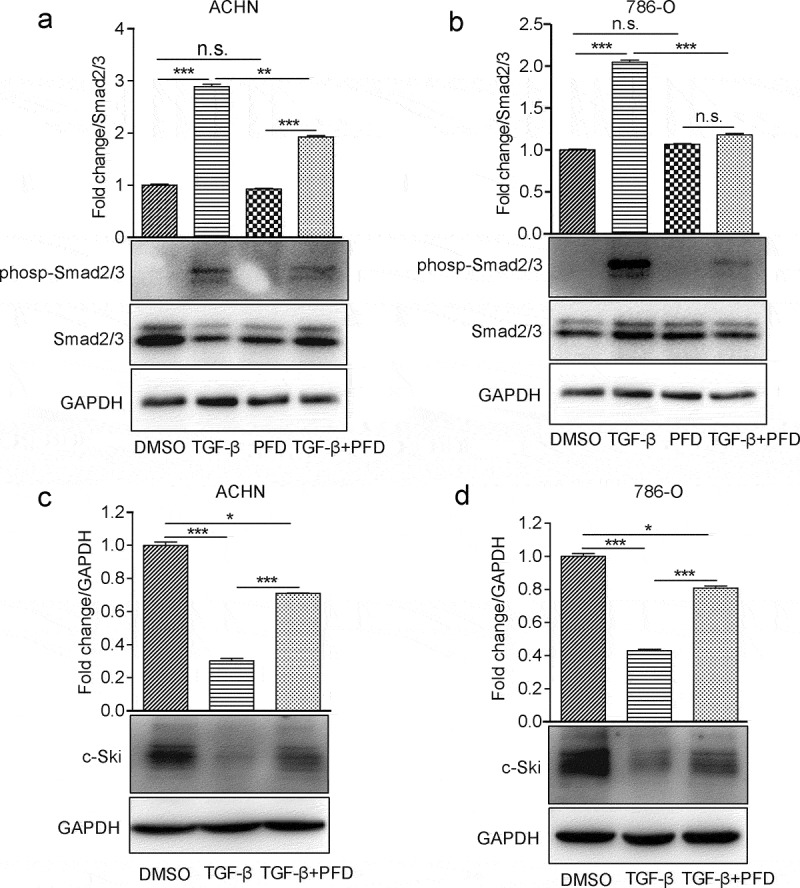
PFD attenuates TGF-β signaling in renal cancer cells.

Next, we examined whether the blockade of TGF-β signaling could benefit the treatment of renal cell carcinoma using PFD, an approved anti-fibrotic drug by US FDA, which is proved to suppress TGF-β signaling in other cells. First, phosphorylated Smad2/3 was significantly increased in the presence of TGF-β, while treatment with PFD inhibited its phosphorylation (Figure 2(a,b)). c-Ski is a reported endogenous TGF-β signaling inhibitor which is degraded by TGF-β before releasing Smad-mediated transcription of downstream targets. In the present study, we found that most of the c-Ski protein was degraded by TGF-β induction and PFD treatment dramatically inhibited TGF-β-induced Ski degradation in renal cancer cells (Figure 2(c,d)). Hence, our results confirmed that PFD could inhibit the biological function of TGF-β for activation of Smad2/3 and degradation of c-Ski in RCC cells.

(A) and (B) PFD suppresses TGF-β-induced Smad phosphorylation. Phosphorylation of Smad2/3 was detected by Western blot in 786-O and ACHN cells in the presence of TGF-β or PFD. (C) and (D) PFD suppresses TGF-β-induced Ski degradation. Western blot analysis for the protein expression of Ski in 786-O and ACHN cells. Data represents the means ± SD. *P < .05; **P < .01; ***P < .001.

**Figure 3.**
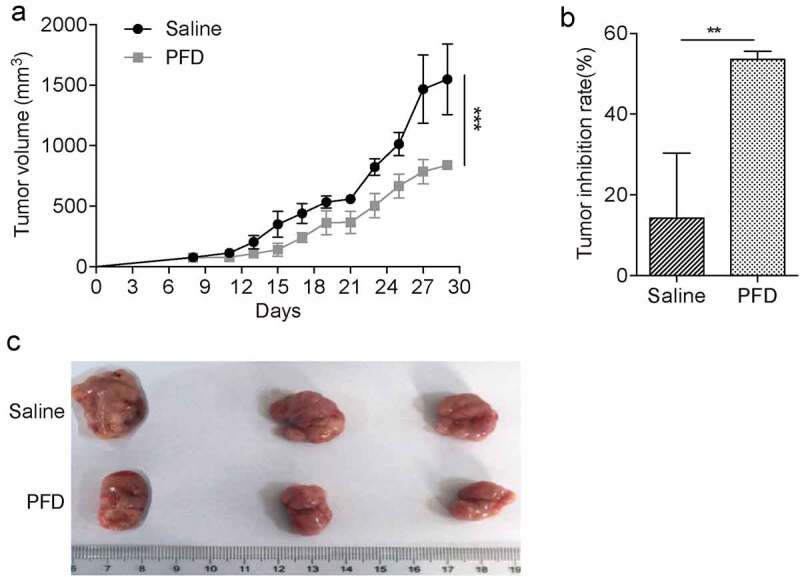
PFD treatment prevents renal cancer progression in mice.

To investigate the therapeutic effects of PFD on renal cell carcinoma *in vivo*, a murine renal cancer cell line Renca-based subcutaneous tumor model was established with immunocompetent Balb/c mice. A week later, when the tumor volume reached 50–100 mm^3^, the mice were randomly grouped and intraperitoneally injected with saline or PFD. We found that tumor growth was significantly prevented after treatment with PFD (Figure 3(a,b,c)). Therefore, the results demonstrate that PFD treatment could suppress renal cell carcinoma progression in the murine model.

Tumor-bearing mice were treated with saline or PFD daily and tumor growth was measured every 3 d until the end of the experiment. (A) Tumor growth curve, (B) tumor inhibition rate were analyzed, and (C) representative tumor pictures were presented to show the therapeutic efficiency of PFD for renal cell carcinoma. **P < .01; ***P < .001.

### 3.3 PFD significantly inhibits proliferation of renal cancer cells in vitro

**Figure 4.**
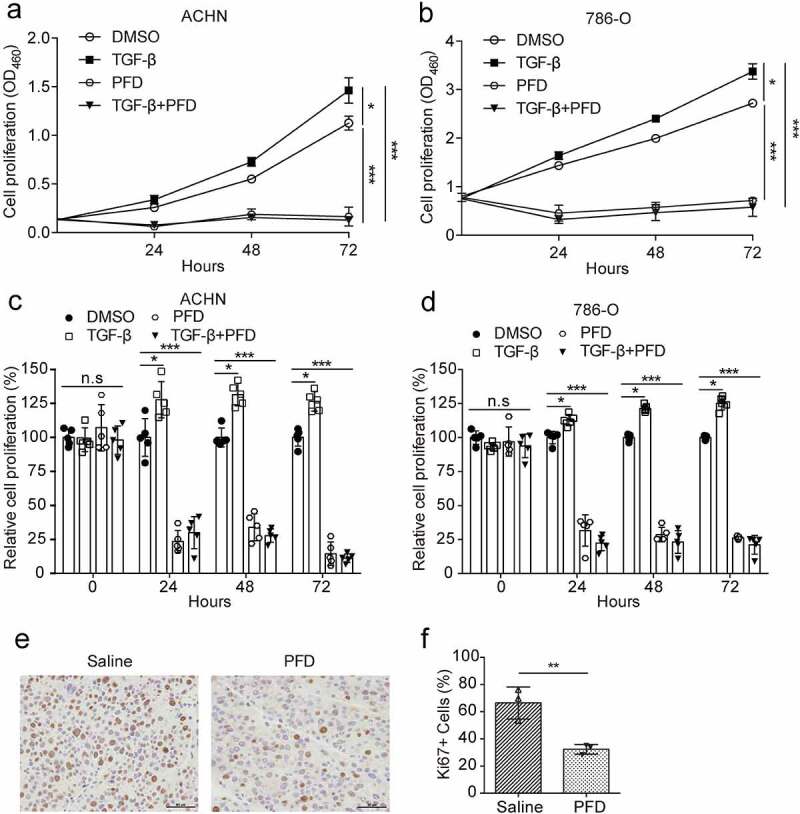
PFD inhibits proliferation of renal cancer cells.

To understand how PFD treatment suppress tumor development, we tested whether PFD treatment inhibits proliferation of renal cancer cells as reported in other cancer types. The optimal dosage, 0.5 mg/ml, of PFD used to treat renal cancer cells was determined by IC50 (data not shown). To detect the proliferation of renal cancer cells, the cell counting kit-8 (CCK-8) assay, a costless and easily performed method without cell toxicity,^31^ was performed. The results showed that a low dose of TGF-β (1 ng/ml) slightly enhanced the proliferation of ACHN and 786-O cells, whereas the presence of PFD at 0.5 mg/ml dramatically inhibited the proliferation of ACHN and 786-O cells and also eliminated TGF-β-stimulated cell proliferation in the time course of 72 h (Figure 4a, B, C, D). Furthermore, Ki67 staining was performed with the paraffin-embedded tumor sections to confirm the proliferative inhibiting efficacy of PFD. The results showed that PFD treatment dramatically decreased the percentage of Ki67-positive hyperproliferative renal cancer cells as compared to saline-treated group (32.34 ± 2.06% versus 66.42 ± 6.79%, Figure 4e, f). Thus, PFD could inhibit the proliferation of renal cancer cells.

(A) and (B) The proliferation of renal cancer cells was performed by CCK-8 assays in 786-O and ACHN cells treated with PFD in the absence or presence of TGF-β for different times (0, 24, 48, 72 h). (C) and (D) The quantification of relative cell proliferation. (E) Representative pictures of Ki67 immunohistochemical staining in paraffin-embedded tumor tissues. (F) Quantification of the percentage of Ki67-positive cells within tumor tissues. Data represents the means ± SD. *P < .05; **P < .01; ***P < .001.

### 3.4 PFD prevents renal cancer cell migration and invasion

**Figure 5.**
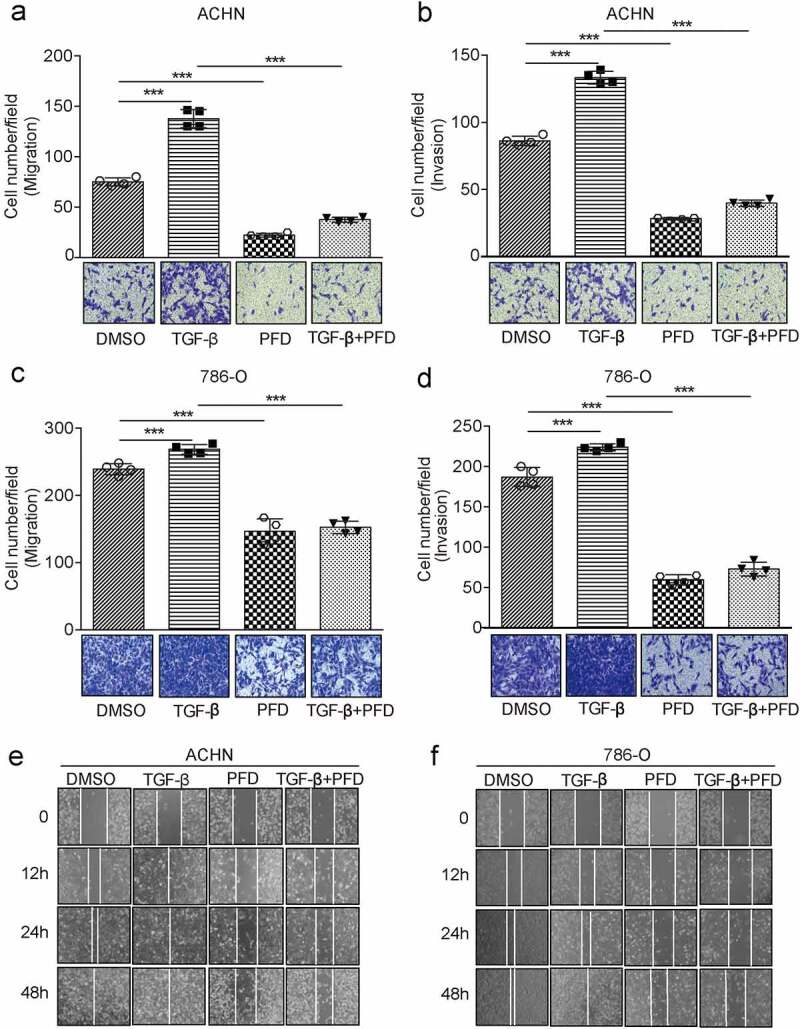
PFD prevents renal cancer cell migration and invasion mediated by TGF-β.

We further investigated the role of PFD in renal cancer cell migration and invasion. The designated amount of 786-O or ACHN cells was inoculated into transwell chambers and treated with DMSO or PFD in the presence of solvent TGF-β. Twenty-four hours later, the transmigrated cells were stained with crystal violet and counted. Results showed that TGF-β promoted migration of ACHN and 786-O by 86% and 12%, respectively. However, treatment with PFD significantly inhibited cell migration by 69% and 33% and even TGF-β-induced cell migration by 49% and 35%, respectively (Figure 5a, b). Regardingthe invasive ability, we found that ACHN and 786-O cells treated with TGF-β were increased by 54% and 22%, while addition of PFD reduced cell invasion by 63% and 70% and also inhibited TGF-β-induced cell invasion by 53% and 59%, respectively (Figure 5c, d).

(A) and (B) The effect of PFD on cell migration and invasion was examined by transwell filter assay in ACHN cells treated with DMSO or PFD in the presence of solvent TGF-β. (C) and (D) The effect of PFD on cell migration and invasion was examined in 786-O cells by transwell filter assay treated with DMSO or PFD in the presence of solvent TGF-β. (E) and (F) The effects of PFD on the motility of ccRCC cells were checked in ACHN and 786-O cells by the wound-healing assay. Data represents the means ± SD. ***P < .001.

To further confirm the inhibiting effect of PFD on renal cell migration, wound-healing assay was performed. As compared to the control group, TGF-β treatment significantly increased the healing rate of cell scratches, while treatment by PFD decreased the healing rate (Figure 5e, f). In conclusion, PFD can inhibit the migration and invasion of renal cancer cells induced by TGF-β.

### 3.5 PFD suppresses TGF-β-induced EMT in renal cancer cells

**Figure 6.**
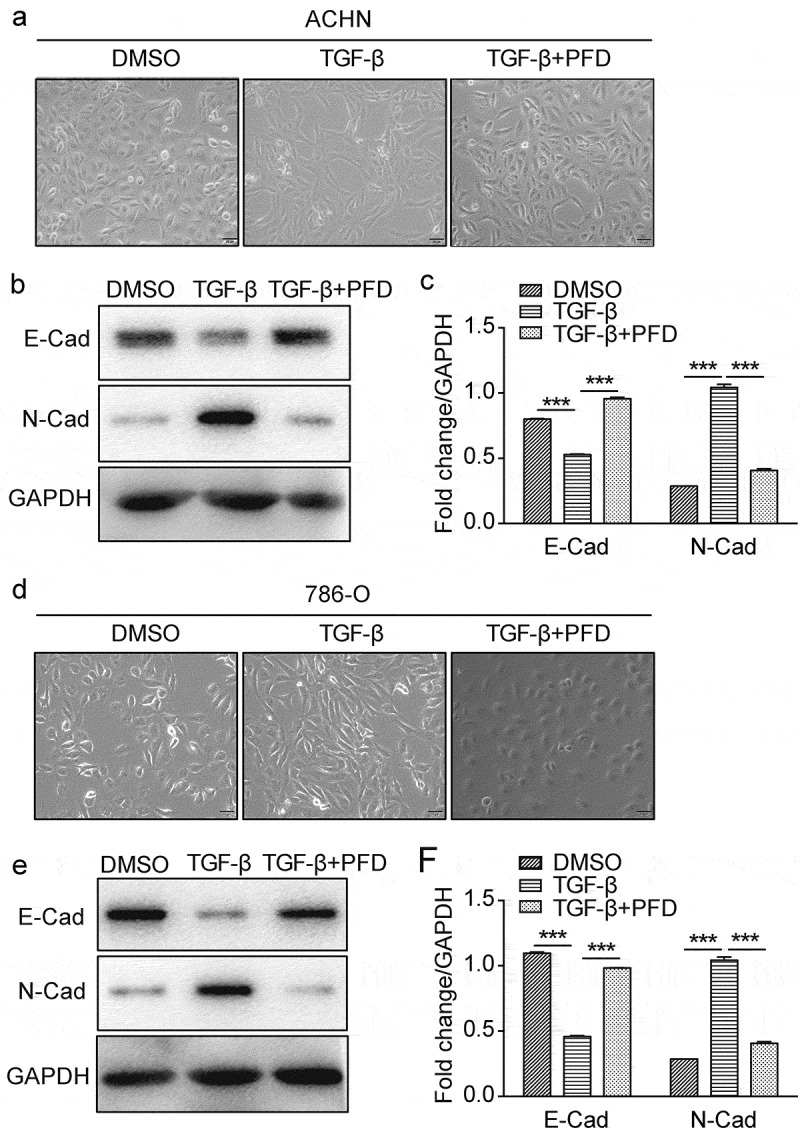
PFD suppresses TGF-β-induced EMT in renal cancer cells.

EMT was reported to play an important role in tumor development and metastasis.^7^ To test whether PFD treatment suppresses TGF-β-induced EMT, we first established the EMT model of renal cancer cells by testing gradient doses of TGF-β and found that 5 ng/ml of TGF-β was optimal for inducing EMT of ACHN and 786-O cells (data not shown). The morphological change of ACHN and 786-O, from cobblestone and epithelial-like to elongated and spindle-like morphology, was observed under the phase-contrast microscope after exposure to TGF-β for 48 h. To our expectation, PFD treatment markedly prevented the morphological change of renal cancer cells induced by TGF-β (Figure 6. a, d). For qualitative detection of EMT morphology, Western blot was carried out to test the protein markers of EMT. We found that EMT was induced successfully regarding the decreased expression of an epithelial marker of E-cadherin and the increased expression of a mesenchymal cell-associated marker of N-cadherin. As predicted, loss of E-cadherin and increase of N-cadherin induced by TGF-β could be reversed by PFD (Figure 6 B,C, E, F). These findings appeared to support that PFD could suppress TGF-β induced EMT in RCC.

(A) and (D) The morphological change of the EMT model of renal cancer cells induced by TGF-β was observed under the phase-contrast microscope after treated with PFD. (B/C) and (E/F) PFD suppresses TGF-β-induced EMT on ACHN and 786-O. Western blot analysis for the protein expression of indicated EMT-related markers (E-cadherin, N-cadherin) in 786-O and ACHN cells. Data represents the means ± SD. ***P < .001.

### 3.6 PFD suppresses autonomous production of TGF-β in RCC cells

**Figure 7.**
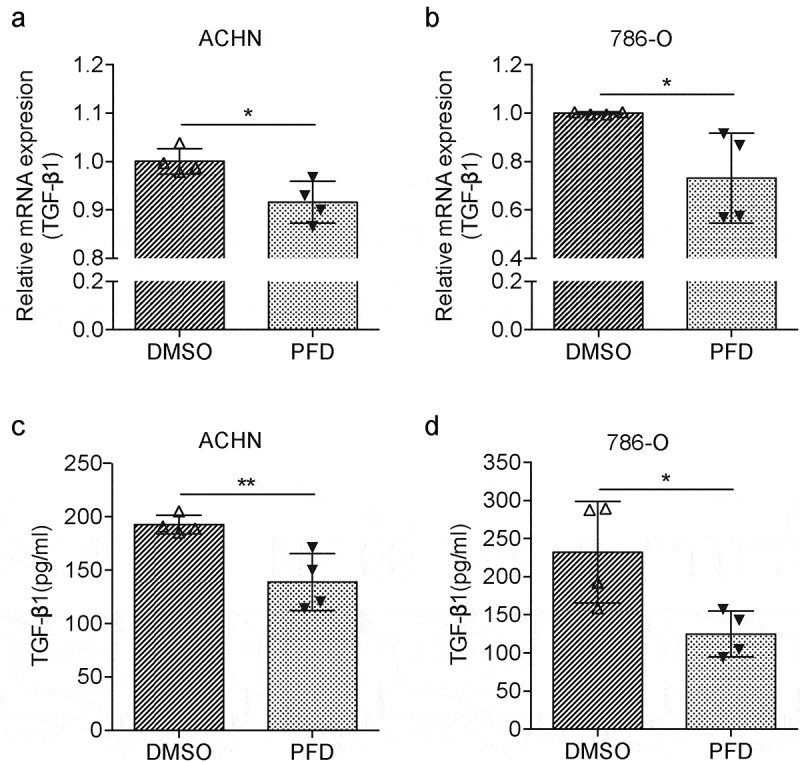
PFD blocks the cell-intrinsic production of TGF-β in RCC cells.

While we found PFD treatment blocks TGF-β signaling in renal cancer cells in the presence of TGF-β, we also noticed that PFD could block tumor cell proliferation (Figure 4), migration, and invasion (Figure 5). These results indicate that PFD may also regulate tumor cell endogenous TGF-β signaling. Indeed, PFD was first reported to suppress TGF-β production when it was used to treat pulmonary or renal fibrosis.^21^ Hence, the expression of TGF-β by renal cancer cells was tested. For mRNA expression assay, 786-O and ACHN cells were collected 24 h later after PFD treatment and total RNA was extracted. The mRNA expression of TGF-β1 was detected by real time quantitative PCR. As compared to the control group, the relative mRNA expression of TGF-β1 in PFD-treated cells was significantly decreased (Figure 7a, b). Furthermore, the protein expression and secretion of TGF-β1 was measured by ELISA. Twenty-four hours after PFD or DMSO treatment, the culture medium of 786-O and ACHN cells was collected for ELISA. Data show that the secreted TGF-β1 was also dramatically downregulated by PFD (Figure 7c, d). In summary, PFD could inhibit the transcription and secretion of TGF-β in renal cancer cells.

(A) and (B) The mRNA expression of TGF-β1 was detected by real-time quantitative PCR in 786-O and ACHN cells after PFD treatment. (C) and (D) The protein expression and secretion of TGF-β1 were measured by ELISA in 786-O and ACHN cells. Data represents the means ± SD. *P < .05; **P < .01.

### 3.7 PFD treatment limits tumor infiltrating MDSCs

**Figure 8.**
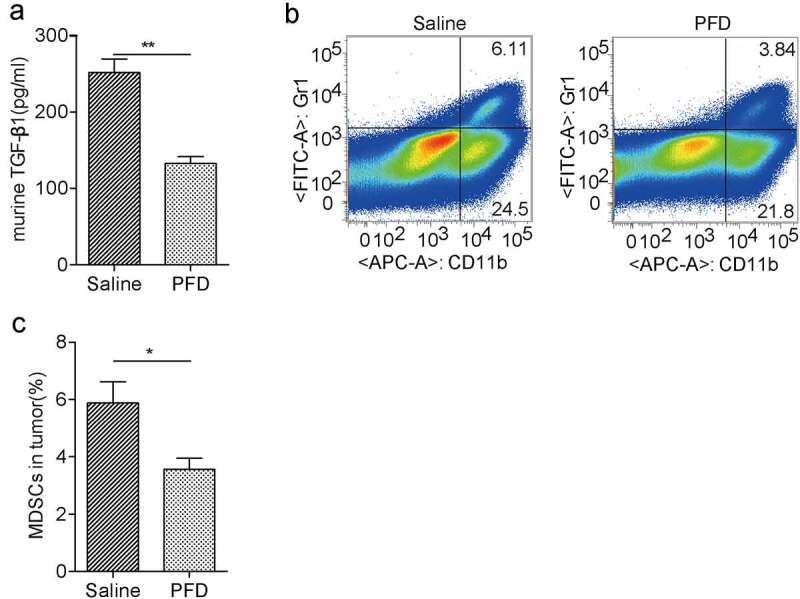
PFD treatment decreases TGF-β production and recruitment of immunosuppressive MDSCs in tumors.

The *in vitro* study prompted us to examine whether PFD also regulates the TGF-β1 expression in tumor-bearing mouse. Indeed, we found serum TGF-β1 from PFD-treated mice was dramatically downregulated as compared to the control group (Figure 8a). As an important modulator, TGF-β contributes to the immunosuppressive tumor microenvironment., We found that the population of tumor-infiltrating MDSCs was significantly decreased in the PFD treatment group compared to the control group (Figure 8b, c). Hence, prevention of renal cancer progression by PFD might be also due to the reduction of TGF-β and immunosuppressive cells except for inhibition of EMT-mediated cancer cell proliferation.

(A) PFD decreases serum TGF-β1 in renal cancer-bearing mice. (B) Flow cytometry analysis and (C) quantification of tumor-infiltrating MDSCs indicate that PFD treatment affects the immunosuppressive tumor microenvironment. Data represents the means ± SD. *P < .05; **P < .01; ***P < .001.

## 4. Discussion

TGF-β is an important cytokine that regulates a variety of biological processes including cell growth, differentiation, apoptosis, immunity, extracellular matrix production, angiogenesis, migration, and invasion.^32,33^ Although TGF-β-induced EMT is a highly complex process that can be mediated by Smad-dependent and -independent signal pathways.^34^ Smad signaling is the major mechanism by which TGF-β induces EMT.^35^ EMT is considered to be an important process for tumor invasion and metastasis.^36^ It has been reported that TGF-β initiates the invasion and metastasis of malignant tumors by inducing EMT in gastric cancer, colorectal cancer, lung cancer, breast cancer, and so on.^10,37–39^ In the present study, we demonstrated that PFD, the anti-fibrotic drug, prevented TGF-β-induced EMT and cell proliferation, cell migration, and invasion of renal cancer cells via blocking Smad2/3 phosphorylation and c-Ski degradation. Therefore, PFD might be a potential adjuvant for treatment of renal cell carcinoma by inhibiting TGF-β signaling.

Except for inhibiting TGF-β-mediated phosphorylation of Smad2/3 by PFD. In the study, we also found that another TGF-β regulator, c-Ski, was affected by PFD treatment. c-Ski was first isolated from chicken embryo and identified as a homolog of the retroviral v-Ski, thus classified as a transforming protein.^40^ The role of c-Ski in tumor progression was controversy. It had been shown to promote tumor progression in leukemia, melanoma, esophageal cancer, colon cancer, and pancreatic cancer^41–45^ and inhibit tumorigenesis in mice^46^ and progression of lung cancer, neuroblastoma.^47,48^ Mechanistically, c-Ski was found binding to Smads and blocking TGF-β-mediated transcription of downstream genes.^49,50^ In turn, studies demonstrated that TGF-β could induce degradation of c-Ski and then release its transcriptional activity.^51,52^ Moreover, silencing of c-Ski promoted TGF-β-induced EMT of cardiomyocytes.^53^ Therefore, PFD prevents renal cancer progression could be due to the inhibition of EMT.

PFD has been verified to have broad effects including anti-fibrotic, anti-inflammatory and antioxidant activity in animal models. It has been approved for the treatment of IPF in Japan, Europe, Canada and the USA as the inhibitor of TGF-β.^54–56^ Many studies have confirmed that PFD plays a synergistic anti-cancer effect in neurofibromatosis, malignant glioma, breast cancer and lung cancer via decreasing TGF-β expression, cancer-associated fibroblasts, tumor microvessel density, etc.^22,23,26,28^ In addition, PFD also has been proved to control tumor growth through promoting infiltration of T cells and NK cells in a murine tumor model.^29,30^ There are 8 registered trials found in NIH ClinicalTrials.gov using PFD to treat advanced-stage NSCLC, neurofibromatosis, uterine leiomyoma, or radiotherapy-induced fibrosis (NCT03177291, NCT00020631, NCT00076102, NCT04243837, NCT04467723, NCT00754780, NCT00053937, NCT00332033). Besides, evidence indicates that PFD protects the kidney from fibrosis and injury induced by ischemia-refusion.^57,58^ Several clinical trials using PFD to treat kidney diseases are undergoing (NCT04258397, NCT04126538, NCT00063583, NCT02530359). Those studies suggest that PFD treatment is beneficial for the recovery of renal function.

In this study, we have demonstrated that PFD can reverse the formation of EMT induced by TGF-β via inhibiting Smad2/3 phosphorylation and c-Ski degradation, thereby alleviating the migration, invasion and proliferation of renal cancer cells induced by TGF-β through a series of experiments, which broaden the potential usage of PFD in antitumoral therapy, especially for renal cancer. Moreover, it is well known that TGF-β plays an immunosuppressive role in the tumor microenvironment by inducing MDSCs. We found PFD treatment did reduce tumor-infiltrating MDSCs, so it might be also exerting its antitumoral effect by modulating the tumor microenvironment. In summary, our findings reveal a new role of PFD in controlling renal cancer and provide a potential therapeutic strategy for renal cell carcinoma.
